# Supplementary material for: Inhibition of Potato Fusarium Wilt by Bacillus subtilis ZWZ-19 and Trichoderma asperellum PT-29: A Comparative Analysis of Non-Targeted Metabolomics
Source: Plants (Basel). 2024 Mar 22;13(7):925. doi: 10.3390/plants13070925 (PMC11013777; doi:10.3390/plants13070925)
Supplement: Supplementary file 1 [file plants-13-00925-s001.zip › Table S1.pdf]

Table S1 Details of all annotated compounds based on LC-MS/MS

Name	Classification	Precursor type	B (AV)	T (AV)	B1T1 (AV)
2-Isopropylmalic acid	Fatty acids and conjugates	[M-H]-	6786916805	87369551	6092473729
Thymine	Organoheterocyclic compounds	[M+H]+	6082210763	166437670.6	8739997.38
2-Naphthol	Benzenoids	[M-H]-	948730.25	9740804.31	5157362
Acetoacetate	Organic acids and derivatives	[M+H]+	22585218.8	276970838.5	111676407.5
2,5-Furandicarboxylic acid	Organoheterocyclic compounds	[M-H]-	30669757.24	246775290.5	302894330.1
Imidazoleacetic acid	Organoheterocyclic compounds	[M-H]-	219579685.2	13398795.5	1691863.927
Itaconic acid	Fatty acids and conjugates	[M-H]-	301088548.5	3896598965	4960172718
Propionylcarnitine	Lipids and lipid-like molecules	[M+H]+	3733976.99	145191887.8	105022418.6
2,3,5,6-Tetramethylpyrazine	Organoheterocyclic compounds	[M+H]+	880563641.8	11894410.09	1835733.72
Adipamide	Lipids and lipid-like molecules	[M+H]+	2668683.65	81594969.91	46801887.98
Isopentenyladenine	Organoheterocyclic compounds	[M+H]+	222086957.1	14490975.9	3195500.92
Hexadecanedioic acid	Lipids and lipid-like molecules	[M-H]-	404915084.4	5191431.81	1215177.15
LPE 14:0	Lipids and lipid-like molecules	[M-H]-	37758216.29	1068933.41	258096.14
Isocitric acid	Organic acids and derivatives	[M-H]-	880563641.8	11894410.09	1835733.72
Ecgonine	Organic acids and derivatives	[M-H]-	1143775758	15494353988	21354922660
Shikimic acid	Organic oxygen compounds	[M-H]-	12654981.66	705598643.4	273575754.7
dAMP	Nucleosides	[M+H]+	640609.82	5388355.48	4688209.17
Kojic acid	Organoheterocyclic compounds	[M+H]+	11834498.58	353340601.3	126988690.5
Dibutyl sebacate	Lipids and lipid-like molecules	[M-H]-	65376909.79	12291093.61	3292017.83
Asaraldehyde	Benzenoids	[M-H]-	4218244.69	1003078177	792333810.6
Indolelactic acid	Organoheterocyclic compounds	[M-H]-	2344453.17	81580571.49	33424052.34
Mesalamine	Benzenoids	[M+H]+	718757.7	7213190	100260977.2
Trigonelline	Others	[M+H]+	200171715	12974513838	4496447369
Acetylcarnitine	Lipids and lipid-like molecules	[M-H]-	235146650.3	7001248.15	3105710.87
5-Aminolevulinic acid	Organic acids and derivatives	[M+H]+	13076926.87	770133463	2739495787
Lipoamide	Organoheterocyclic compounds	[M-H]-	52730373.34	3557355.95	1139368.74

Histamine	Amines	[M+H]+	8154162.72	91916102.61	38534769.19
D-Xylonic Acid	Organic oxygen compounds	[M-H]-	1093617180	8075843221	3972602199
P-Mentha-1,3,8-triene	Lipids and lipid-like molecules	[M+H]+	1163617.27	23256076.74	8794846.09
Tetradecanedioic acid	Lipids and lipid-like molecules	[M-H]-	155249033.4	8693640.18	2992118.43
N-Oleoyl Glycine	Organoheterocyclic compounds	[M+H]+	86049922.88	3130745.73	790282.13
11β-Hydroxyandrosterone	Lipids and lipid-like molecules	[M+H]+	86238132.48	25428354.76	8410186.78
Uric acid	Organoheterocyclic compounds	[M-H]-	39154410.53	7001859.21	3150517.80
Meclocycline	Others	[M+H]+	7409268.34	1266537.86	578518.41
Phytosphingosine	Amines	[M+H]+	8649895.43	439525002.6	482137680.3
L-Alanyl-L-proline	Amino acids	[M+H]+	9644259.95	108179105	91593361.49
5'-Adenylic acid	Nucleosides	[M+H]+	7978742.11	14773138.33	12548217.13
N-Acetyl-L-aspartic acid	amino acids	[M-H]-	86049922.88	3130745.73	790282.13
4-Hydroxyphenylacetic acid	Benzenoids	[M+H]+	15689796.69	1300517998	444539228.8
N-Methylanthranilic acid	Benzenoids	[M+H]+	132253167.9	959865748.3	446531127.2
Gentisic acid	Benzenoids	[M-H]-	30590729.65	4299131.99	1558452.89
Paracetamol	Benzenoids	[M+H]+	75280578.74	16753226704	8141003376
Myristic acid	Lipids and lipid-like molecules	[M-H]-	4469585.14	1873381.21	346159.31
Perillartine	Lipids and lipid-like molecules	[M+H]+	122961256.3	6745974.76	3884225.94
Glycerol 1-hexadecanoate	Lipids and lipid-like molecules	[M+H]+	72696942.13	9471879.38	3282026.28
2-Furoic acid	Organoheterocyclic compounds	[M-H]-	14547786.28	2086045197	914987491
Orotic acid	Organoheterocyclic compounds	[M-H]-	177427785.7	8159165.30	4897971.96
Dithranol	Benzenoids	[M+H]+	507211.91	8180584.44	2425293.91
L-(-)-Arabitol	Others	[M-H]-	117042628.6	24502063.41	9864546.58
4-Phenylbutyric acid	amino acid	[M-H]-	6148693.35	7304127339	3394217379
Abametapir	Organoheterocyclic compounds	[M+H]+	1008282.57	398901586.8	111036941.5
Lactitol	Lipids and lipid-like molecules	[M-H]-	1773039.70	18664907.1	6260225.6
Benzamidine	Benzenoids	[M+H]+	2598912.31	88865187.22	22455647.93
Edaravone	Organoheterocyclic compounds	[M+H]+	4677771.72	80174008.59	23906077.6

1-Methyladenine	Organoheterocyclic compounds	[M+H]+	73843413.74	8329093.10	4527502.79
2-Oxindole	Organoheterocyclic compounds	[M+H]+	34406599.85	7 555653495.5	223010384.8
13-HPODE	Lipids and lipid-like molecules	[M-H]-	10965553.71	85357561.46	73411383.98
Norephedrine	Benzenoids	[M+H]+	79972053.69	61731532.31	5123352.76
Glutaconic acid	Amino acids	[M-H]-	949798311.1	14983974136	6898838428
S-Adenosyl-methionine	Nucleosides	[M+H]+	68391722.95	5852865.74	3149717.75
Hordenine	Benzenoids	[M+H]+	2652570.79	9.1E+09	5.39E+09
Trans,trans-2,4-Heptadienal	Others	[M-H]-	5332138.59	4.3E+09	5.15E+09
N-acetyl-D-glucosamine	Others	[M-H]-	53680705.75	1.2E+08	2.48E+08
D-Ala-D-Ala	Amino acids	[M-H]-	2276486.32	5077339.04	10299978.01
Gamma-Glutamylleucine	Amino acids	[M-H]-	36091740.97	4841473.61	8601215.23
D-glutamine	Amino acids	[M+H]+	2670126.29	3385520.74	3311855.45
Corticosterone	Lipids and lipid-like molecules	[M-H]-	209410.13	56774566.38	1210983.32
Palmitoyl-Sn-Glycero-3-Phosphocholine	Lipids and lipid-like molecules	[M+H]+	10559038.59	4409318.84	8436475.89
Phe-Phe	Amino acids	[M+H]+	17780361.92	12286.91	24051321.95
Trans-Aconitic acid	Amino acids	[M-H]-	1.67E+08	1.24E+09	3.22E+09
α-Aspartylphenylalanine	Amino acids	[M-H]-	3303102.32	4389128.04	6670298.65
Ala-Leu	Amino acids	[M-H]-	32078576.46	34990251.84	71148766.94
Xanthosine	Nucleosides	[M-H]-	1.49E+08	1.86E+08	2.19E+08
O-Phospho-L-Tyrosine	Amino acids	[M+H]+	8682204.22	50112828.93	79985442.23
Prolylleucine	Amino acids	[M+H]+	1661250.38	28523571.39	4.89E+08
Taurocholic acid	Lipids and lipid-like molecules	[M-H]-	776887.04	1.81E+08	951002.95
Tauroursodeoxycholic acid	Lipids and lipid-like molecules	[M-H]-	222166.72	11915542.98	226330.52
Nicotinuric Acid	Amino acids	[M+H]+	2475960.22	10315495.88	38998552.72
Tyrosylalanine	Amino acids	[M+H]+	6123169.88	12543444.03	13078256.47
L-Leucyl-L-Alanine	Amino acids	[M-H]-	11105654.91	3272512.55	15614296.71
D-Glucaric acid	Others	[M-H]-	3.37E+09	6.95E+09	1.79E+10
2-Methylbutyroylcarnitine	Lipids and lipid-like molecules	[M-H]-	66430117.53	1173388.70	5432422.73

Thr-Leu	Amino acids	[M+H]+	34792745.72	12556604.24	41012613.87
Uracil	Organoheterocyclic compounds	[M-H]-	1.74E+08	13915870.47	1.49E+08
Leucylproline	Amino acids	[M+H]+	2189690.63	54001053.08	1.15E+09
Ascorbyl palmitate	Lipids and lipid-like molecules	[M-H]-	290040.8	367096	375595.5
Phenylacetylglutamine	Amino acids	[M-H]-	3552767.91	5963652.76	3815441.15
4-Ethylphenol	Benzenoids	[M-H]-	38958572.37	34615164.2	10425920.13
L-Asparagine	Amino acids	[M-H]-	100765.82	1094561.11	510346.29
Methyl nicotinate	Organoheterocyclic compounds	[M+H]+	8091893.92	63149670.94	21217432.8
L-Methionine sulfoxide	Amino acids	[M+H]+	18836149.48	25703143.55	10294681.92
L-Tryptophan	Organoheterocyclic compounds	[M+H]+	3.70E+08	1.46E+09	6.88E+08
UDP-galactose	Nucleosides	[M-H]-	776677.24	11525086.91	15830670.16
L-Tyrosine	Amino acids	[M+H]+	1.55E+09	8.60E+08	1.32E+09
8-Aminooctanoic acid	amino acid	[M-H]-	14283785.99	15507276.09	3986557.13
3,4-Dihydroxyphenylacetic acid	Benzenoids	[M-H]-	700360.9	919207.8	2.21E+08
Pyrogallol	Benzenoids	[M+H]+	1.21E+08	2.2E+08	98605027.59
1,3-Dimethyluracil	Organoheterocyclic compounds	[M+H]+	541865.25	5936451.72	2760712.18
LL-2,6-Diaminopimelate	Amino acids	[M-H]-	14124537.98	919684.27	1593870.33
4-Methoxycinnamic Acid	Others	[M-H]-	41007573.5	86042543.53	19122129.35
5-Phenylvaleric Acid	Lipids and lipid-like molecules	[M-H]-	1141679.19	99528331.14	11902138.47
Diisobutyl adipate	Lipids and lipid-like molecules	[M+H]+	85774439.56	1.77E+08	34249374.02
N-Acetyl-L-tyrosine	Amino acids	[M+H]+	19215603.86	1.95E+08	54893850.74
Isotretinoin	Organoheterocyclic compounds	[M+H]+	2238895.04	3.42E+08	74066609.89
9-Aminoacridine	Organoheterocyclic compounds	[M+H]+	11660744.81	56753852.14	13771707.78
L-Malate	Amino acids	[M-H]-	16565672.05	6474048.41	1593721.02
Acetophenone	Others	[M+H]+	15359706.15	11076216.93	5822024.28
4-Ethylbenzaldehyde	Benzenoids	[M+H]+	28447705.77	83722183.77	10493278.03
2-(Formylamino)Benzoic Acid	Benzenoids	[M-H]-	36205503.99	1.63E+08	62378037.19
Pyridoxamine	Organoheterocyclic compounds	[M+H]+	9604931.03	13054976.29	5392172.08

Urocanic acid	Organoheterocyclic compounds	[M+H]+	8.46E+08	72959539.78	2.78E+08
Gly-Phe	Amino acids	[M-H]-	23254459.5	17806193.6	7938105.94
Asp-glu	Amino acids	[M+H]+	23773591.96	20198030.71	1.41E+08
D-myo-Inositol 1,4-bisphosphate	Others	[M-H]-	4799500.86	10017555.94	5469883.19
5-Methylcytosine	Organoheterocyclic compounds	[M+H]+	13446988.74	2.66E+08	61196280.31
Suberic acid	Lipids and lipid-like molecules	[M-H]-	17127329.2	37242439.95	22722641.53
17alpha-Hydroxyprogesterone	Lipids and lipid-like molecules	[M+H]+	265730.78	685751.32	350368.01
Xanthine	Organoheterocyclic compounds	[M+H]+	69086820.22	1.82E+08	1.78E+08
2-Oxoglutaric acid	Others	[M-H]-	47415017.22	4.32E+08	1.04E+08
Nonadecanoic acid	Lipids and lipid-like molecules	[M-H]-	1.80E+08	811796.74	859458.62
2-Hydroxycaproic acid	Lipids and lipid-like molecules	[M-H]-	1.29E+09	1.63E+09	4.88E+08
2-Hydroxyphenylalanine	Amino acids	[M+H]+	77476049.67	2.30E+08	70809105.6
N-acetyl-L-ornithine	Amino acids	[M+H]+	2174910.99	4647655.77	2870512.80
D-Threose	Others	[M-H]-	8.92E+08	6.32E+09	3.67E+09
Tropine	Others	[M+H]+	2.57E+08	6.24E+08	1.97E+08
3,4-Dimethylbenzoic acid	Benzenoids	[M+H]+	5589226.27	14703515.54	4318089.01
Deoxyguanosine	Nucleosides	[M+H]+	2910276.50	60883504.53	29332969.56
8,15-Dihete	Lipids and lipid-like molecules	[M+H]+	3022536.98	123980640.1	40044358.12
Indole-3-butyric acid	Organoheterocyclic compounds	[M-H]-	6121762.66	92117409.68	20964658
1-Stearoylglycerol	Lipids and lipid-like molecules	[M+H]+	6.28E+08	1.77E+09	1.38E+09
Cortodoxone	Lipids and lipid-like molecules	[M+H]+	2472711.11	2.67E+08	3605352.9
Deoxynivalenol	Lipids and lipid-like molecules	[M+H]+	4263932.04	3.09E+08	31192535.67
Sucrose	Others	[M-H]-	2.28E+08	3.39E+09	6.80E+08
Maltotriose	Others	[M-H]-	2.20E+09	4.00E+09	2.29E+09

AV means average value.
