# Supplementary material for: Inhibition of Potato Fusarium Wilt by Bacillus subtilis ZWZ-19 and Trichoderma asperellum PT-29: A Comparative Analysis of Non-Targeted Metabolomics
Source: Plants (Basel). 2024 Mar 22;13(7):925. doi: 10.3390/plants13070925 (PMC11013777; doi:10.3390/plants13070925)
Supplement: Supplementary file 1 [file plants-13-00925-s001.zip › Table S2.pdf]

Table S2 Significantly changed KEGG pathways

Pathway	Total	Hits	-LOG(p)	Impactor	Association with biocontrol mechanisms
**Inositol phosphate metabolism**	8	2	1.1506	1	Involving in cell signaling, like synthesis of cell walls, jasmonic acid signaling, and auxin regulation etc.
**Taurine and hypotaurine metabolism**	10	2	0.31318	0.85714	Not reported
**Pyruvate metabolism**	26	6	0.832	0.4133	Inducing plant resistance to pathogen infection，like Inducing resistance of sunflower to verticillium wilt by Pyruvate
**Citrate cycle (TCA cycle)**	20	6	0.87154	0.29662	Inhibition of pathogenic bacteria, like citric acid on spoilage bacteria of deible mushroom
**Alanine, aspartate and glutamate metabolism**	22	7	1.0119	0.28846	Inhibition of pathogenic bacteria, like the inhibitory activity of copper-aspartate complexes on Erwinia amylovora
**Glycine, serine and threonine metabolism**	33	5	0.17921	0.27546	Enhancing the competitiveness of biocontrol bacteria and pathogens, like Being strengthened the competitiveness with the pathogenic bacteria by Valine-glycine protein from Lysobacter capsica X2-3
**Butanoate metabolism**	17	3	1.3277	0.23809	Not reported

**Nicotinate and nicotinamide metabolism**	15	2	1.8432	0.22137	Not reported
**Pentose phosphate pathway**	26	2	0.8772	0.2157	Not reported
**Glycolysis / Gluconeogenesis**	29	4	1.1903	0.20568	Not reported

Total, the total number of metabolites in the target metabolic pathway; Hits, the number of differential metabolites in the target metabolic pathway; −log (p): −log(p-value); impact, the greater the effect of metabolic pathways, the better
