# Supplementary figures and images for: Inhibition of Potato Fusarium Wilt by Bacillus subtilis ZWZ-19 and Trichoderma asperellum PT-29: A Comparative Analysis of Non-Targeted Metabolomics
Source: Plants (Basel). 2024 Mar 22;13(7):925. doi: 10.3390/plants13070925 (PMC11013777; doi:10.3390/plants13070925)

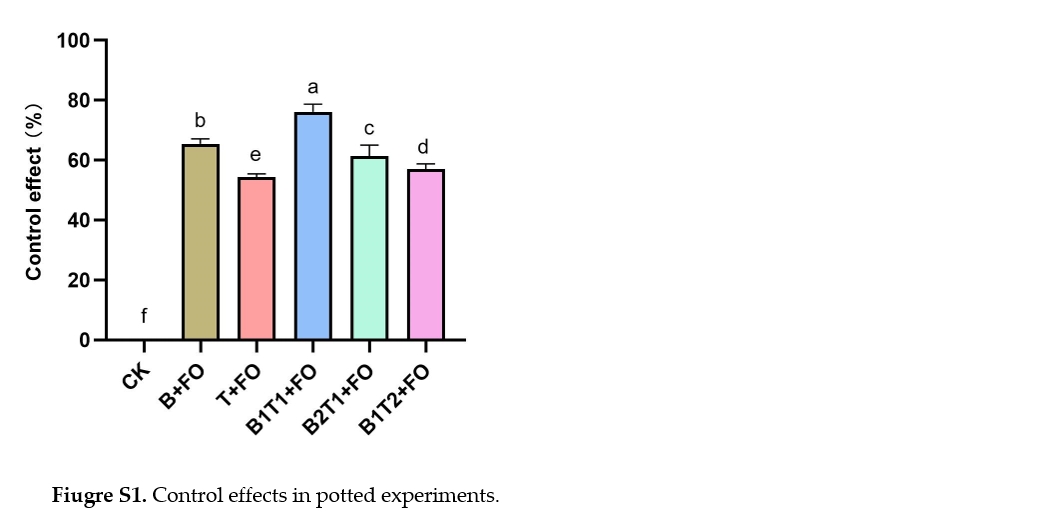

Supplement: Supplementary file 1 [file plants-13-00925-s001.zip › Figure S1.tif]
